# CAFU: a Galaxy framework for exploring unmapped RNA-Seq data

**DOI:** 10.1093/bib/bbz018

**Published:** 2019-02-28

**Authors:** Siyuan Chen, Chengzhi Ren, Jingjing Zhai, Jiantao Yu, Xuyang Zhao, Zelong Li, Ting Zhang, Wenlong Ma, Zhaoxue Han, Chuang Ma

**Affiliations:** 1 State Key Laboratory of Crop Stress Biology for Arid Areas, Center of Bioinformatics, College of Life Sciences, Northwest Agriculture and Forestry University; 2 College of Information Engineering, Northwest Agriculture and Forestry University

**Keywords:** Galaxy, pipeline, machine learning, RNA-Seq, unmapped reads, workflow

## Abstract

A widely used approach in transcriptome analysis is the alignment of short reads to a reference genome. However, owing to the deficiencies of specially designed analytical systems, short reads unmapped to the genome sequence are usually ignored, resulting in the loss of significant biological information and insights. To fill this gap, we present Comprehensive Assembly and Functional annotation of Unmapped RNA-Seq data (CAFU), a Galaxy-based framework that can facilitate the large-scale analysis of unmapped RNA sequencing (RNA-Seq) reads from single- and mixed-species samples. By taking advantage of machine learning techniques, CAFU addresses the issue of accurately identifying the species origin of transcripts assembled using unmapped reads from mixed-species samples. CAFU also represents an innovation in that it provides a comprehensive collection of functions required for transcript confidence evaluation, coding potential calculation, sequence and expression characterization and function annotation. These functions and their dependencies have been integrated into a Galaxy framework that provides access to CAFU via a user-friendly interface, dramatically simplifying complex exploration tasks involving unmapped RNA-Seq reads. CAFU has been validated with RNA-Seq data sets from wheat and *Zea mays* (maize) samples. CAFU is freely available via GitHub: https://github.com/cma2015/CAFU.

## Introduction

Rapid advances in next-generation sequencing (NGS) technologies have enabled us to decipher the genomes of both model and non-model species, providing multi-layered omics data at a reasonable cost [1, 2]. At the level of transcriptomics, millions of short reads (usually 100–150 base pairs) generated from RNA sequencing (RNA-Seq) provide valuable resources for investigating the structure and dynamics of genes under defined conditions. In most workflows, short RNA-Seq reads are analyzed based on their alignments with the genome sequence. However, in such analysis, a small but significant fraction of RNA-Seq reads is usually unexplored, owing to their unmappability to the genome sequence. Unmapped RNA-Seq reads may be caused by several factors, including the incompleteness of genome sequences, the inherent limitations of alignment programs and the sequencing of mixed-species (e.g. pathogen–host) samples [3–7].

In recent years, the importance of unmapped RNA-Seq reads has been widely recognized [4, 8–11]. A large-scale analysis of unmapped RNA-Seq reads from >17 000 human disease-related samples identified reads from archaeal, bacterial or viral genomes, highlighting the role of the microbiome in human disease [4]. Unmapped RNA-Seq reads are also valuable resources to identify novel transcripts missing from the existing genome annotation. For example, Kazemian *et al.* identified 2550 novel human transcripts from ~300 million unmapped RNA-Seq reads from 11 normal and 21 cancer tissues [10]. Assembled transcripts from unmapped RNA-Seq reads offer researchers an opportunity to identify novel transcripts associated with specific cancers in humans [10] and with agricultural traits in maize [9]. Such surveys indicate that ignoring unmapped reads may lead to the loss of important biological information in RNA-Seq data analysis for many organisms.

Many frameworks have been developed for mapped reads; however, until now, none has been specially designed for the comprehensive analysis of unmapped reads (Supplementary Data Table S1). One obstacle is that the majority of existing NGS programs are not user friendly, are complicated or require extensive preparation steps. Another is that most analysis (e.g. parameter optimization and sequential implementation) requires researchers to program custom scripts, which can result in errors that affect reproducibility. Finally, specialist software is required to deeply mine unmapped RNA-Seq reads, especially for those from mixed-species samples generated by dual RNA-Seq experiments. Dual RNA-Seq simultaneously profiles the transcriptomes of the pathogen and the host in mixed-species samples and has been a powerful tool in the study of pathogen–host interactions [12]. Thus, there is a need to develop a program to accurately determine the species of origin of transcripts assembled using unmapped RNA-Seq reads from mixed-species samples. In our experience, the large-scale exploration of unmapped RNA-Seq data presents a considerable challenge for many researchers.

Here, we present an analytical framework and accompanying web-based Galaxy platform for comprehensive assembly and functional annotation of unmapped RNA-Seq data (CAFU) from single- and mixed-species samples. CAFU not only facilitates basic analysis of RNA-Seq reads, including read cleansing and mapping, unmapped read extraction and *de novo* transcription assembly, but also introduces several novel functions. Taking advantage of machine learning (ML) technologies, CAFU addresses the challenge of identifying the species of origin of transcripts assembled using unmapped reads from mixed-species samples. Furthermore, CAFU offers multiple-level evidence evaluation, sequence and expression characterization and transcript function annotation. We have demonstrated the effectiveness of CAFU in the analysis of unmapped RNA-Seq reads in wheat and maize. To enhance the application of CAFU, all functions and their dependencies have been combined into a Galaxy platform and further packaged into a Docker image (~14 GB). Through standardized packaging techniques, comprehensive user documents, detailed case studies and wiki discussion groups at the webpage of the CAFU project (https://github.com/cma2015/CAFU), we aim to ensure that researchers, regardless of their informatics expertise, can benefit from our framework for accessible, reproducible and collaborative analysis of large-volume unmapped RNA-Seq data.

## Materials and methods

### Overview of the CAFU framework

**Figure 1:**
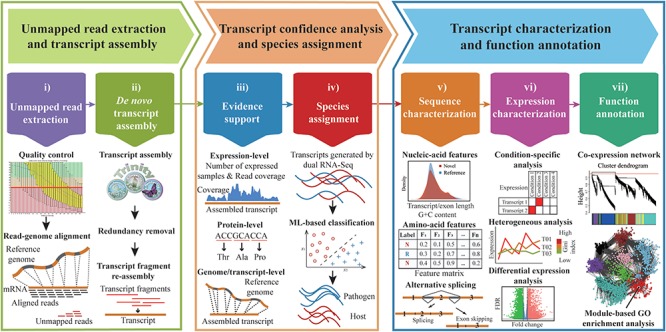
Overview of CAFU.

The Galaxy-based framework, CAFU, is composed of 7 modules, covering 17 functions, developed with existing NGS tools as well as a set of programs developed by ourselves (Figure 1; Supplementary Data Table S2). The details of these functional modules are presented in the following.

#### Extraction of unmapped reads

To run CAFU, users will typically start with a set of RNA-Seq data and genome sequences. The quality of RNA-Seq data is first examined using FastQC [13], followed by trimming of poly-A/T sequences and low-quality bases using fqtrim [14] and Trimmomatic [15]. After trimming, reads shorter than a specified length (e.g. 20 bp) are also discarded. The remaining reads are subsequently mapped to the genome sequences using the fast, splice-aware alignment program HISAT2 [16], yielding a Sequence Alignment Map (SAM) file recording read-genome alignments. Unmapped reads (paired-end reads in which both ends are unmapped and single-end reads, which are unmapped) are extracted from the SAM file by using SAMTools [17] and BEDTools [18]. Specifically, for RNA-Seq reads from mixed-species (e.g. pathogen–host) samples, CAFU first aligns RNA-Seq reads against the host genome sequences. The resulting unmapped reads are then aligned against the pathogen genome sequences. After this two-step read-genome alignment, reads unmapped to genome sequences of both species are output in fastq format. This process of generation of unmapped reads is iteratively performed for all RNA-Seq data from different samples.

As unmapped reads may result from contamination during sampling or RNA-Seq, CAFU also provides options to remove potential contamination sequences using Deconseq [19] with user-specific matching coverage and identity (e.g. 0.95). A built-in database, which included 3529 bacterial reference genomes (as of 5 November 2018) and 81 viral reference genomes (as of 5 November 2018) from the National Center for Biotechnology Information (NCBI), was provided for users to remove contamination. Likewise, users can also submit customized contamination sequences.

#### *De novo* transcript assembly of unmapped reads

Unmapped reads from different samples are pooled together and used as an input to Trinity [20] to generate transcript fragments through *de novo* assembly. To ensure that assembled transcripts are more ‘complete’, transcript fragments are input into CD-HIT-EST [21] to reduce redundancy with a sequence identity cutoff (e.g. 0.90). The nonredundant transcript fragments are further merged to generate longer transcripts using CAP3 [22]. Two fragments are merged if they meet the criteria of a specified overlap size (e.g. ≥50 bp) and identity (e.g. ≥98%).

#### Multiple-level evidence analysis of assembled transcripts

To eliminate possible artifacts introduced by *de novo* transcript assembly, CAFU provides evidence of assembled transcripts at the expression, genome, transcript and protein levels.

(a) The expression-level evidence allows users to eliminate assembled transcripts with low read coverage and/or low expression abundance, which are likely to be assembly artifacts. RNA-Seq reads from different samples are mapped to newly assembled transcripts and reference transcripts using bowtie2 [23]. CAFU outputs the read coverage of assembled transcripts at single-base resolution using BEDTools [18] and estimates the expression abundance of all transcripts in terms of fragments per kilobase million (FPKM) using RSEM [24]. Assembled transcripts with low read coverage (e.g. <10) or low expression (e.g. FPKM <1) in the majority of samples (e.g. 80%) are discarded.
(b) The genome-level evidence can be used to identify *de novo*-assembled transcripts missing from the existing genome annotation. CAFU aligns assembled transcripts to the genome sequences of the corresponding and closely related species using GMAP [25] and selects the best genomic matches with high identity (e.g. ≥95%) and coverage (e.g. ≥95%). Users can also eliminate assembled transcripts with no introns, which could represent either noise or pseudogenes.
(c) The transcript-level evidence can be used to select assembled transcripts with high similarity to other well-annotated transcripts, such as full-length transcripts generated from single-molecule real-time sequencing and/or high-quality transcripts annotated in closely related species. After aligning assembled transcripts with other well-annotated transcripts with GMAP, CAFU outputs the best transcript alignments with high identity (e.g. ≥95%) and coverage (e.g. ≥95%).
(d) The protein-level evidence indicates whether or not an assembled transcript can be translated into a protein. CAFU assesses the coding potential of assembled transcripts using CPC2 [26], which is a fast and accurate coding potential calculator built with ML algorithms and sequence intrinsic features. Assembled transcripts are regarded as coding transcripts if they have a coding potential score ≥0.5 and a specific amino acid length (e.g. ≥100). Otherwise, assembled transcripts are regarded as noncoding transcripts. For coding transcripts, putative domains in corresponding protein sequences are identified using the Pfam database [27].

#### Species assignment of assembled transcripts

**Figure 2:**
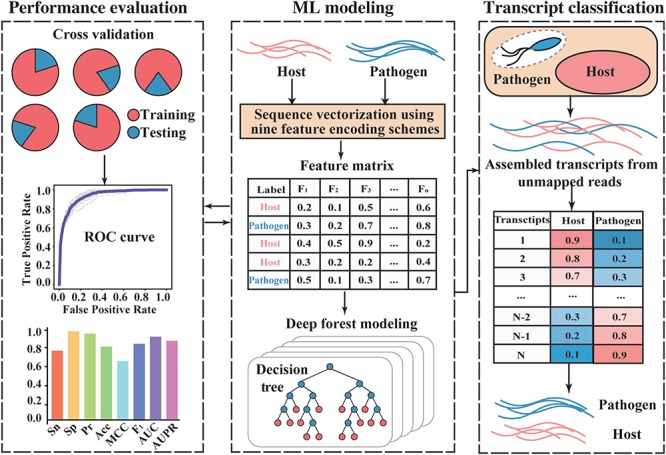
Overview of SAT functional module in CAFU.

This module is specifically designed for coding transcripts assembled using unmapped reads from mixed-species samples. Existing coding potential calculators, such as CPC2 [26] and CPAT [28], have good capability for distinguishing protein-coding transcripts from noncoding transcripts in many species. However, they are often species-neutral and do not detect information regarding the original species of coding transcripts, resulting in difficulties in exploring pathogen–host interactions from unmapped RNA-Seq reads. To address this problem, we developed an ML-based functional module named species assignment of transcripts (SAT) to pinpoint the species categories of assembled transcripts, based on features extracted from amino acid sequences of pathogen and host species (Figure 2).

As the input, SAT takes the coding sequences of pathogen and host reference transcripts. Each sequence is first converted into a fixed-length (2257-dimensional) numeric vector using nine feature-encoding schemes (see Supplementary Data for details): k-mer (420 features), distance-based residues (DR, 1220 features), autocovariance (6 features), cross-covariance (CC, 12 features), auto-CC (ACC, 18 features), physicochemical distance transformation (531 features), series correlation pseudo acid composition (PC-PseAAC, 22 features), general series correlation PseAAC, (26 features) and codon usage bias (CUB, two features). Then, the *M* (number of sequences) × *N* (number of features, 2257) feature matrix is fed into the deep forest algorithm [29], which is a decision tree-based ensemble learning method with the complexity of a deep neural network but without hyperparameter tuning. Next, a predictor for classifying assembled transcripts is constructed, the performance of which is evaluated using a 5-fold cross-validation approach with different evaluation measures, including the receiver operating characteristic (ROC) curve, precision-recall (PR) curve, sensitivity (Sn), specificity (Sp), precision, accuracy (Acc), Matthews correlation coefficient and F_1_-score. Finally, SAT assigns a probability score to each tested sequence, indicating the likelihood that the transcript belongs to the pathogen or host species.

#### Sequence characterization of assembled transcripts

(a) Basic character: users can explore the similarity between assembled and reference transcripts in terms of the distribution of transcript length and G+C content, as well as amino acid-based features used in SAT. The significance level of the similarity between two distributions is estimated using the Kolmogorov–Smirnov test.
(b) Alternative splicing for assembled transcripts is explored using the R package SGSeq [30].

#### Expression characterization of assembled transcripts

The distribution of expression levels of all transcripts under different experimental conditions can be characterized through condition-specificity analysis, heterogeneous analysis and differential expression (DE) analysis.

(a) Condition-specificity analysis: this analysis identifies a set of transcripts highly expressed under different conditions. The condition specificity of a transcript for condition type *T* is defined using the formula described in [31]: *CS*(*i*) = 1 −$\frac{median_{x\in \overline{S}}{E}_i^x}{median_{x\in S}{E}_i^x}$, where ${median}_{x\in S}{E}_i^x$ and ${median}_{x\in \overline{S}}{E}_i^x$ represent the median expression values of transcript *i* under experimental condition *T* and under other experimental conditions, respectively. That is, the higher the condition-specific score of a transcript under one experimental condition, the more likely the transcript is to be specifically expressed under this experimental condition.
(b) Heter ogeneous analysis: this analysis examines the stability of each transcript expressed in all samples using the Gini index (coefficient), which is widely used by economists to investigate inequalities in wealth distribution in populations [32]. Gini index values range from 0 (full equality) to 1 (extreme inequality); a low value indicates that the transcript is stably expressed and may be a housekeeping transcript [33].
(c) DE analysis: DE transcripts are identified using EBSeq [34], with a suitable fold change (e.g. ≥2.0) and false discovery rate (FDR)-adjusted *P*-value (e.g. ≤0.05).

#### Function annotation of assembled transcripts

The potential functions of assembled transcripts are explored using weighted gene co-expression network analysis [35], a systems biology method for gene function analysis that groups transcripts with similar expression patterns into one module [36–39]. The transcript expression similarity is calculated using the Gini correlation coefficient [40]. Gene Ontology (GO) enrichment analysis of each module is performed using topGO [41].

### Framework efficiency

CAFU has been used to analyze unmapped RNA-Seq reads from wheat and maize samples on an Intel(R) Xeon(R) E5-2678 v3 48-core machine with 2.50 GHz speed and 132 GB RAM. Thirty Xiaoyan 6 (XY 6) wheat paired-end RNA-Seq samples were downloaded from the NCBI’s Sequence Read Archive database under accession number PRJNA387101; 171 maize paired-end RNA-Seq samples were collected from the NCBI BioProject repository under accession numbers PRJNA171684, PRJNA237837 and PRJNA272662; and 94 maize drought-related RNA-Seq samples were obtained from BioProject under accession number PRJNA291919. The costs in terms of time and computer resources for each functional module are shown in Supplementary Data Table S3. A subset of newly assembled transcripts was experimentally validated using polymerase chain reaction (PCR) and sequencing. More details about the experimental validation (plant material preparation, RNA isolation and cDNA synthesis, PCR amplification and sequencing) can be found in the Supplementary Data.

## Results

### Application of CAFU to unmapped RNA-Seq reads in wheat

**Figure 3:**
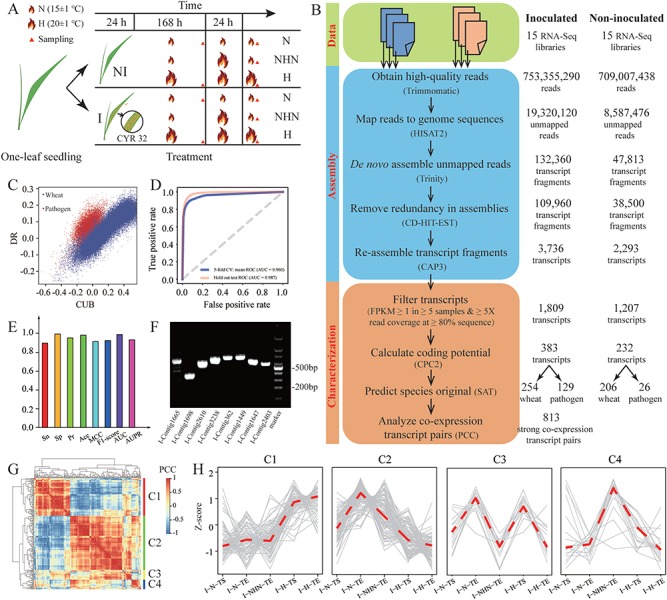
Application of CAFU to unmapped RNA-Seq reads from stripe rust-infected and uninfected wheat samples. **(A)** shows the experimental process of obtaining 30 RNA-Seq data from wheat seedlings under different inoculation and temperature treatments. **(B)** Data mining of unmapped RNA-Seq reads for identifying wheat and pathogen transcripts. **(C)** Dot plot of CUB and DR; blue and red dots denote wheat and pathogen mRNAs, respectively. **(D)** ROC curves of 5-fold cross-validation for SAT functional module. The diagonal line is a reference representing 0.5 AUC. **(E)** Performance evaluation of SAT using hold-out testing samples. **(F)** PCR amplification of four predicted wheat transcripts (I-Contig1665, I-Contig1698, I-Contig2610 and I-Contig3238) and four predicted pathogen transcripts (I-Contig362, I-Contig1449, I-Contig1647 and I-Contig2403). cDNAs for the PCR amplification of wheat and pathogen transcripts were prepared from XY 6 wheat seedlings and stripe rust-infected wheat seedlings, respectively. **(G)** Heat map of Pearson’s correlations between 216 assembled transcripts. **(H)** Expression patterns of assembled transcripts in four clusters. Gray lines represent Z-score-normalized expression levels of all transcripts in the corresponding cluster, and red lines represent the median value of normalized expression levels.

We first demonstrated CAFU’s utility by exploring unmapped reads from 15 stripe rust-infected and 15 uninfected wheat RNA-Seq samples (Supplementary Data Table S4). More details regarding these samples can be found in [42]. Briefly, wheat (XY 6) seedlings were inoculated with Chinese yellow rust race 32 (CYR32), which is one of the most frequent and virulent races among the identified stripe rust pathogens [43]. Then wheat seedlings with (I) and without (NI) inoculation were further grown under three different temperature conditions, normal temperature (N; 15 ± 1°C), heat stress (H; 20 ± 1°C) and NHN (first grown at 15 ± 1°C for 7 days, then transferred to 20 ± 1°C for 24 hours and finally moved back to 15 ± 1°C), and harvested at the start (TS) and end (TE) points of temperature treatment (Figure 3A). Finally, inoculated wheat samples (I-N-TS, I-N-TE, I-NHN-TE, I-H-TS and I-H-TE; each for three biological replicates) and noninoculated wheat samples (NI-N-TS, NI-N-TE, NI-NHN-TE, NI-H-TS and NI-H-TE; each for three biological replicates) were subjected to RNA-Seq to generate 101 bp paired-end reads, using the Illumina HiSeq 2000 platform.

After trimming sequencing adapters and low-quality reads, ~1.46 billion clean reads were first mapped to the reference genome of Chinese Spring wheat (*Triticum aestivum* L.; https://plants.ensembl.org/Triticum_aestivum). Unmapped reads were then mapped to the reference genome of stripe rust pathogen *(Puccinia striiformis f. sp. Tritici* PST 78; https://fungi.ensembl.org/Puccinia_striiformis_f_sp_tritici_pst_78)*.* As a result, we obtained a total of 27.91 million unmapped reads (14.42% per sample on average). For noninoculated and inoculated wheat samples, the corresponding unmapped reads were assembled into 1207 and 1809 transcripts, respectively, which were expressed in at least 5 samples (FPKM ≥1) and had at least 5× read coverage across at least 80% of the transcript sequence. We observed that >74% unmapped reads can be aligned to assembled transcripts. That is to say, >74% unmapped reads analyzed in this study could be reused by CAFU. Further, CPC2 analysis indicated that 232 and 383 putatively coding transcripts could be obtained from the unmapped reads for the noninoculated and inoculated wheat samples, respectively (Figure 3B; Supplementary Data Table S5). A total of 50.86% (118/232) transcripts from noninoculated wheat samples and 58.22% (223/383) transcripts from inoculated wheat samples can be annotated by Pfam database (Supplementary Data Table S5). Several transcripts assembled from inoculated wheat samples may play roles in disease resistance. For example, I-Contig2176 encodes an aci-reductone-dioxygenase (ARD) domain-containing protein. It has >98% sequence identity with the protein sequence of *TaARD* gene, which has been reported to be responsive to stripe rust pathogen infection in wheat [44]; I-Contig159 encodes an amidase domain-containing protein, which has ~87% identity with protein XP_020192680.1 [fatty acid amide hydrolase-like (AtFAAH) (*Aegilops tauschii subsp. tauschii*)]. It has reported that overexpression of AtFAAH compromises innate immunity to bacterial pathogens in *Arabidopsis* [45]; I-Contig2155 has a fairly high identity (99.15%) and the same ICL (isocitrate lyase) family domain with protein BAI66426.1 [ICL (*Triticum aestivum*)]; ICL and malate synthase (MS) are unique enzymes of glyoxylate cycle; recent studies indicate that ICL and MS play important roles in human, animal and plant pathogenesis [46].

To identify the original species of the coding transcripts from wheat and stripe rust pathogen, the SAT module in CAFU was trained using coding regions of 20 502 and 137 052 mRNAs annotated in the reference genome of stripe rust pathogen *Puccinia striiformis f. sp. tritici* (PST-78 v1) and Chinese Spring wheat (IWGSC RefSeq v1.0), respectively (see Supplementary Data for details). To explore the performance of SAT, we plotted two major features (DR and CUB) in a two-dimensional space and observed that mRNAs from wheat and stripe rust were grouped into two distinct clusters (Figure 3C). This indicated that the features used in SAT had enough discriminative power for transcript classification. As expected, 5-fold cross-validation experimental results showed that CAFU had a promising prediction performance, with an area under the ROC curve (AUC) of 0.960, in the classification of mRNAs from stripe rust and wheat (Figure 3D; Supplementary Data). The high performance of CAFU was also demonstrated on the hold-out testing data set, with an AUC of 0.987 (Figure 3D–E) and area under PR (AUPR) curve of 0.933. Meanwhile, with a threshold cutoff of 0.5, CAFU generated an Acc of 94.1%, Sn of 97.6% and Sp of 93.8% (Figure 3E). We further applied CAFU to identify the species of origin of assembled transcripts and found that the majority of assembled transcripts (206/232) using unmapped reads from noninoculated samples were predicted to be wheat transcripts (Supplementary Data Table S5). For the 383 assembled transcripts using unmapped reads from inoculation samples, 254 were predicted to be wheat transcripts (score ≥0.5) and the other 129 assembled transcripts were predicted to be pathogen transcripts. Four wheat transcripts and four pathogen transcripts were randomly selected and experimentally validated by PCR amplification (Figure 3F; Supplementary Data Figure S1; Supplementary Data Table S6).

The heterogeneous analysis showed that 216 of 383 transcripts assembled using unmapped reads from the inoculated wheat samples had varying expression levels (Gini index ≥0.1) among the 5 experimental conditions (I-N-TS, I-N-TE, I-NHN-TE, I-H-TS and I-H-TE). Pearson correlation coefficient (PCC) and hierarchical clustering analysis revealed that these 216 assembled transcripts could be grouped into four major clusters (Figure 3G), each of which was composed of predicted wheat and pathogen transcripts with similar expression patterns across the five experimental conditions (Figure 3H). We also observed that 813 transcript pairs exhibited strong co-expression relationships (|PCC| ≥ 0.90). These results indicate that the mining of unmapped RNA-Seq reads could be used to identify novel transcripts and co-expression relationships, allowing researchers to generate a more comprehensive picture of transcript co-expression for resolving host–pathogen interactions.

**Figure 4:**
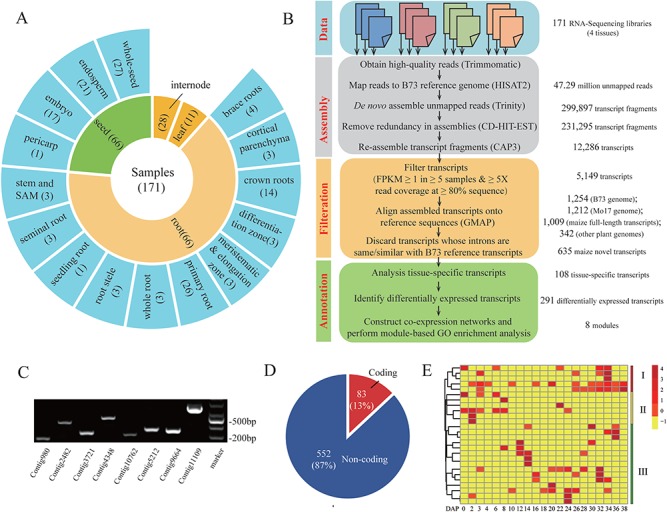
Application of CAFU to unmapped RNA-Seq reads from maize B73 samples. **(A)** provides tissue information for 171 RNA-Seq samples. The numbers of samples are in parentheses. **(B)** Data processing strategy for identifying maize transcripts from unmapped RNA-Seq reads. **(C)** PCR amplification of eight newly assembled maize transcripts using cDNAs from B73 seedling samples. **(D)** shows the number of different coding potential transcripts. **(E)** shows the expression heat map of seed-specific maize transcripts at different seed development stages.

### Application of CAFU to unmapped RNA-Seq reads in maize

We further applied CAFU to explore unmapped RNA-Seq reads from 171 maize B73 samples (Figure 4A-4B; Supplementary Data Table S7). Approximately 4.71 billion clean reads were aligned to the maize B73 reference genome (APGv4); the quality and coverage of which were recently significantly improved by single-molecule real-time sequencing and high-resolution optical mapping [47]. Despite improvements in the alignment methods and maize reference genome, ~50 million reads remained unmapped to the maize reference genome (Supplementary Data Table S7). The unmapped reads were *de novo* assembled into 12 286 long transcripts, 5419 of which were expressed with FPKM ≥1 in at least 5 samples and had at least 5× read coverage across at least 80% of the transcript sequence (Supplementary Data Table S8). This analysis results to the reuse of >54% unmapped reads analyzed in this study. Using the criteria of ≥95% coverage and identity, 1254 of these 5419 assembled transcripts could be aligned onto the maize B73 reference genome (Supplementary Data Table S8). From this, we could identify 581 novel transcripts that were missing in the maize B73 reference genome annotation but were found in the unmapped reads (Supplementary Data Table S9). We found that 550 of these 581 newly discovered transcripts had high sequence similarity (≥95% coverage and identity) with the maize full-length single-molecule sequencing transcripts (437 transcripts) [48] and/or the reference genome sequences of maize Mo17 (517 transcripts), *Sorghum bicolor* (73 transcripts), *Oryza sativa* L. ssp. *japonica* (1 transcript), *Setaria italica* (6 transcripts), *Brachypodium distachyon* (1 transcript) and *Arabidopsis thaliana* (1 transcript; Supplementary Data Table S9). We found that 54 assembled transcripts had no hits or relatively low sequence similarity (<95% coverage and/or identity) with the maize B73 reference genome; however, they were matched (≥95% coverage and identity) to the maize B73 single-molecule sequencing transcription and reference genome of the plant species under study (Supplementary Data Table S9)*.* We randomly selected eight transcripts with significant matches to the maize B73 reference genome, maize Mo17 reference genome, *Sorghum bicolor* genome and/or full-length single-molecule sequencing transcripts and experimentally validated them by PCR amplification using specific primers (Supplementary Data Table S10), followed by Sanger sequencing from a mixture of cDNA libraries derived from well-watered and drought-stressed maize seedling tissues (Figure 4C; Supplementary Data Figure S2). These results indicate that the novel transcripts, regardless of whether they matched the corresponding reference genome, could be identified from unmapped RNA-Seq data.

The downstream analysis focused on the 635 (581 + 54) assembled transcripts that had strong evidence support at the genome and/or transcript level. The length of these 635 novel assembled transcripts (mean ± SD, 514 ± 497 bp) was much shorter than the length of maize transcripts annotated in the Ensembl Plants database (mean ± SD, 2600 ± 1631 bp). Using the coding potential calculator CPC2, 83 of these 635 novel transcripts were classified as protein-coding RNAs (Figure 4D; Supplementary Data Table S9), including putative transcription factors containing bZIP and zf-C2H2 domains.

We next evaluated whether any of our newly discovered maize transcripts had putative biological function. The expression levels of all newly assembled and reference transcripts were first estimated using all 171 RNA-Seq libraries. Then transcripts highly expressed in the same tissues were identified using a tissue-specific score threshold of 0.8. In seed tissue, we detected 40 tissue-specific transcripts assembled from unmapped RNA-Seq reads (Figure 4E). The hierarchical clustering analysis revealed that these transcripts could be divided into three groups with temporal patterns during maize seed development from 0 to 38 days after pollination (Figure 4E). Five transcripts (group I) exhibited higher expression levels in both the early and later stages of whole seed development than in the middle stages, including one transcript (Conitg1938) encoding a trehalose-phosphatase domain-containing protein. In *Arabidopsis,* a member of the trehalose-phosphatase domain gene family may be involved in seed maturation and germination [49]. Six transcripts (group II) showed relatively high expression levels in the early stage of whole seed, while 15 transcripts (group III) exhibited higher expression levels in the middle stage. These results indicate potential roles for these seed-specific novel transcripts during maize seed development.

**Figure 5:**
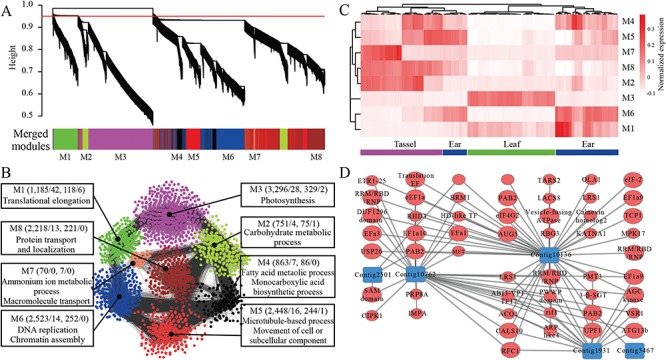
Functional characterization of differentially expressed transcripts. **(A)** Hierarchical cluster tree showing co-expression modules identified using weighted gene co-expression network analysis. **(B)** GO enrichments of eight modules in the co-expression network. **(C)** shows the expression heat map of transcripts in different modules. **(D)** Subnetwork showing connections between newly assembled transcripts and reference transcripts encoding translation-related proteins. The subnetwork was constructed using 58 transcripts with top-100 highest correlation coefficients.

We next explored whether any of the newly assembled maize transcripts were involved in drought stress. Using 94 drought stress-related RNA-Seq libraries [50], we estimated the expression abundance of newly assembled and reference transcripts in 3 tissues of maize that spanned 4 developmental stages (V12, V14, V18 and R1) under well-watered and drought stress conditions. This resulted in the identification of 291 assembled transcripts and 54 484 reference transcripts that showed significant expression changes (fold change ≥2.0 and FDR-adjusted *P* ≤ 0.05). The biological functions of these DE transcripts were further explored using a co-expression network approach [35]. The co-expression network was constructed using 13 354 DE transcripts (including 124 novel transcripts) that expressed more than a third of the samples in 94 libraries with a coefficient of variation greater than one (Supplementary Data Table S11). A total of 14 modules were identified according to the hierarchical clustering results. Highly correlated clusters of these 14 modules were then merged using the ‘mergeCloseModules’ function with cutHeight set to 0.15 to generate 8 modules (Figure 5A). In these 8 modules, the number of co-expressed transcripts ranged from 70 to 3296 (Figure 5B). Transcripts in the same module displayed similar expression trends across diverse conditions (Figure 5C); thus, functional coherence among the transcripts in the same module is expected. GO enrichment analysis showed that each module had distinct GO terms (Figure 5B; Supplementary Data Table S12). Module M1 formed a cluster of 1185 transcripts enriched in translation (Figure 5B). Hierarchical clustering of module eigengenes showed higher expression in ear and leaf tissues (Figure 5C). Tissue-specific transcript expression was observed in modules M2, M3, M7 and M8, which are associated with photosynthesis and macromolecule transport (Figure 5B and C); 3296 transcripts including 28 novel transcripts in M3 were related to photosynthesis and were highly expressed in leaf tissues. In module M6, 2523 transcripts including 14 novel transcripts were associated with DNA replication and chromatin assembly; the expression of these transcripts was observed in four ear development stages and some tassel development stages (Figure 5B and C).

For each of these eight modules, intramodular hub transcripts were identified based on the correlation with module eigengenes. According to this, transcripts could be grouped into two classes: hub transcripts and non-hub transcripts. In total, 10 novel transcripts distributed in M1, M2, M3 and M5 were identified as hub transcripts (Figure 5B). Module M3, which is a photosynthesis-related cluster, contained 329 hub transcripts, including two novel hub transcripts: Contig461 and Contig5212. Contig5212 showed a high correlation (Spearman correlation coefficient, 0.96) with reference transcript Zm00001d042840_T001, which may be involved in the pathway of carbohydrate biosynthesis. Module M1, which is associated with the translation process, included 118 hub transcripts (including 6 novel transcripts: Contig1931, Contig2501, Contig5419, Contig5467, Contig10136 and Contig10762). Contig1931, Contig10136 and Contig10762 were differentially expressed in the drought-stress response in ear and leaf tissues of maize (Supplementary Data Table S11). We visualized a subnetwork using 58 transcripts with top 100 highest correlation coefficients. Among these 58 transcripts, there were 5 novel transcripts (Contig2501, Contig10762, Contig10136, Contig1931 and Contig5467) and several transcripts encoding translation factors (e.g. eEF1a9 [Zm00001d046449_T015], eEF1a10[Zm00001d036904_T013] and eIF4G2[Zm00001d025777_T006]; Figure 5D).

## Discussion

RNA-Seq, a revolution methodology for RNA profiling based on NGS, has been widely applied in both model and non-model plant species, altering our view of the extent and complexity of transcriptomics during the development of plants and animals and under different experimental conditions. Despite its successes, challenges associated with large-scale RNA-Seq data analysis remain. One key challenge is the deep mining of biological knowledge from unmapped reads, which are usually considered to be noise or contamination and therefore are generally ignored [6–8]. The large-scale nature of RNA-Seq data, the incompleteness and inaccuracy of genome sequences, the fast-evolving and command line-based nature of the computational tools available and the lack of a unified pipeline deter biologists from taking part in the processing and analysis of unmapped reads. To help address this challenge, this work presented a Galaxy-based system CAFU with a user-friendly interface to facilitate the comprehensive assembly and functional annotation of unmapped RNA-Seq reads. Compared with the existing aligned reads analysis pipeline, CAFU has several advantages.

First, CAFU is compatible with the analysis of large-scale unmapped reads from traditional and dual RNA-Seq experiments. Traditional RNA-Seq analysis typically focuses on transcriptomes from a single species at a time. An iterative process can be performed to process unmapped reads from different samples. Given the increased volume and depth of sequencing that is now available, dual RNA-Seq experiments can be used to simultaneously profile gene expression in multiple species (e.g. pathogen and host) from mixed-species (infected) samples, providing further insights into host–pathogen interactions that currently cannot be obtained by sequencing of the individual players. After aligning RNA-Seq data against the respective host and pathogen genome sequences, reads mapped to either the pathogen or the host genome are usually used for quantification and function analysis. As further complementary work, our framework CAFU not only performs *de novo* assembly of transcripts from unmapped reads from mixed-species samples but also takes advantage of ML technologies to assign original species of assembled transcripts, based on discriminative properties of mRNAs in these two different domains of life (pathogen and host). Thus, CAFU is expected to be valuable in obtaining a more complete picture of pathogen–host infection.

Second, CAFU offers multiple functionalities. It contains a comprehensive collection of functions required for quality control, removal of low-quality reads and *de novo* assembly of unmapped reads. CAFU also provides options to explore evidence of assembled transcripts at the expression, genome, transcript and protein levels, guiding users to select assembled transcripts of interest for downstream analysis. Additionally, CAFU allows users to characterize newly assembled transcripts at the sequence and expression levels and to explore their functions through gene co-expression analysis. These functionalities can also benefit bioinformaticians as they can be integrated with other Galaxy-based NGS analysis platforms, such as Rnnotator [51], Eoulsan [52] and Oqtans [53].

Third, CAFU is user friendly. By taking advantage of the Galaxy platform, CAFU provides an easy-to-use interface with functions that allow users to configure the implementation of the different functionalities, manipulate large-scale RNA-Seq data, set different parameters, examine the running status and visualize the computational outputs of multiple steps. Users can customize the workflow execution by selecting appropriate functional modules and tuning corresponding parameters according to the data set at hand. In order to facilitate nonexpert users in their analyses, we also provide a set of default parameters derived from our own analysis experience. To address the issues of data security, data sharing and high-performance computing, we have made CAFU available via a Docker image, in which all computational programs, newly developed scripts and dependencies are packaged. This modern packaging strategy overcomes issues related to code changes, dependencies and backward compatibility over time. The easy implementation of CAFU, as well as detailed case studies, comprehensive explanations of the input and output and wiki discussion groups, supports users throughout their work and thus lowers the barriers for researchers unfamiliar with specific NGS data analyses.

Considering the broad applications of RNA-Seq in life science communities, CAFU is potentially broadly applicable to the in-depth study of unmapped reads across plant, animal and microbial species. To facilitate its utility, the CAFU project is hosted on GitHub and is available for download at https://github.com/cma2015/CAFU.

## Supplementary Material

Supp_bbz018

## Notes

Key Points

- RNA-Seq is a powerful tool to study transcriptome characteristics in both model and non-model species. RNA-Seq data are generally analyzed by aligning short reads to genome sequences. Unmapped RNA-Seq reads are usually discarded from the analysis process, resulting in a loss of significant biological information and insights.
- A modularized unmapped RNA-Seq data processing pipeline is proposed in this work, covering a series of general RNA-Seq data analytical functionalities, as well as several specifically designed functionalities.
- Comprehensive Assembly and Functional annotation of Unmapped RNA-Seq data (CAFU) takes advantage of machine learning technologies to identify the species of origin of transcripts assembled using unmapped RNA-Seq reads from mixed-species samples.
- CAFU provides a convenient framework for researchers to thoroughly explore unmapped RNA-Seq reads using the Galaxy system.

## Notes

**Siyuan Chen** is a PhD candidate from Ma’s Laboratory. His research interests include the development of bioinformatics workflows and software for the analysis of multiomic data.

## Notes

**Chengzhi Ren** is a master student from Ma’s Laboratory. His current research theme is plant molecular biology and biochemistry.

## Notes

**Jingjing Zhai** is a master student from Ma’s Laboratory. Her research includes machine learning (ML)-based omics data analysis.

## Notes

**Jiantao Yu** is a lecturer at the College of Information Engineering, Northwest Agriculture and Forestry (A&F) University, with expertise in bioinformatics and cloud-based high-throughput computing.

## Notes

**Xuyang Zhao** is an undergraduate student at the College of Information Engineering, Northwest A&F University. His current research focuses on cloud-based omics data analysis.

## Notes

**Zelong Li** is a master student from Ma’s Laboratory. His current research theme is plant molecular biology and biochemistry.

## Notes

**Ting Zhang** is a PhD candidate from Ma’s Laboratory. His research interests include biological network development and analysis.

## Notes

**Wenlong Ma** is a PhD candidate from Ma’s Laboratory. His research interests include ML-based omics data analysis.

## Notes

**Zhaoxue Han** is an associate professor from Ma’s Laboratory. Her research interests include plant molecular biology and biochemistry.

## Notes

**Chuang Ma** is the director of the Bioinformatics Laboratory of Northwest A&F University and a professor of bioinformatics at the State Key Laboratory of Crop Stress Biology for Arid Areas, Center of Bioinformatics, College of Life Sciences, Northwest A&F University and at the Key Laboratory of Biology and Genetics Improvement of Maize in Arid Area of Northwest Region, Ministry of Agriculture, Northwest A&F University, Shaanxi, China.
