# Supplementary material for: CAFU: a Galaxy framework for exploring unmapped RNA-Seq data
Source: Brief Bioinform. 2019 Feb 28;21(2):676–86. doi: 10.1093/bib/bbz018 (PMC7299299; doi:10.1093/bib/bbz018)
Supplement: Supp_bbz018 [file supp_bbz018.docx]

Supplementary Materials

**CAFU: A Galaxy framework for exploring unmapped RNA-Seq data**

Siyuan Chen^*^, Chengzhi Ren^*^, Jingjing Zhai^*^, Jiantao Yu^*^, Xuyang Zhao, Zelong Li, Ting Zhang, Wenlong Ma, Zhaoxue Han, Chuang Ma

Corresponding Author: Chuang Ma, State Key Laboratory of Crop Stress Biology for Arid Areas, Center of Bioinformatics, College of Life Sciences, Northwest A&F University, Shaanxi, Yangling 712100, China. Tel.: +86-29-87091109; Email: chuangma2006@gmail.com; cma@nwafu.edu.cn

*These authors contributed equally to this work.

**Supplementary Figures and Tables are available online at the website of the CAFU project (https://github.com/cma2015/CAFU).**

**Supplementary Table Legends**

**Supplementary Table S1**. Selected list of RNA-Seq analysis pipelines.

**Supplementary Table S2**. Overview of functional modules in CAFU.

**Supplementary Table S3**. Time costs and computer resources for exploring wheat and maize RNA-Seq data.

**Supplementary Table S4**. List of wheat RNA-Seq data and corresponding mapping statistics.

**Supplementary Table S5**. Wheat and pathogen transcripts assembled from unmapped RNA-Seq reads.

**Supplementary Table S6**. Primer sequences of wheat and pathogen transcripts used for experimental validation.

**Supplementary Table S7**. List of maize RNA-seq data and corresponding mapping statistics.

**Supplementary Table S8**. Sequences of 5,149 transcripts assembled from unmapped RNA-Seq reads.

**Supplementary Table S9**. Evidence support of newly assembled transcripts at the genome and/or transcript levels.

**Supplementary Table S10**. Primer sequences of eight maize transcripts used for experimental validation.

**Supplementary Table S11**. Differential expression and network module information of maize transcripts.

**Supplementary Table S12**. GO enrichment results of each module in the co-expression network.

**Supplementary Figure Legends**

**Supplementary Figure S1**. PCR amplification and sequencing of eight randomly selected transcripts assembled using unmapped RNA-Seq reads from inoculation and non-inoculation wheat samples.

**Supplementary Figure S2**. PCR amplification and sequencing of eight randomly selected transcripts assembled using unmapped RNA-Seq reads from 171 maize B73 samples.

**Supplementary Documents**

**1. Species assignment of newly assembled transcripts**

In CAFU, we introduce SAT (Species Assignment of Transcripts), a machine learning (ML)-based functional module that pinpoints the species categories of assembled transcripts. Below we present a case study of SAT in identifying the original species of transcripts assembled from unmapped reads from stripe rust (*Puccinia striiformis f. sp. tritici*)-infected wheat samples.

- 1. **Positive and negative samples**

The SAT was trained and validated using 20,502 positive samples and 137,052 negative samples. The positive samples were coding sequences of *Puccinia striiformis f. sp. tritici* (PST-78) mRNAs obtained from Ensembl Fungi database (https://fungi.ensembl.org/Puccinia_striiformis_f_sp_tritici_pst_78), while the negative samples were coding sequences of bread wheat (*Triticum aestivum* L.) mRNAs obtained from the Ensembl Plants database (https://plants.ensembl.org/index.html). These positive and negative samples were randomly divided into two parts: 80% training samples (16,401 positive and 109,641 negative samples) and 20% hold-out testing samples (4,101 positive and 27,411 negative samples).

- 1. **Feature encoding**

In order to be recognized by the ML system, each sample was represented to a fixed-length numeric vector by nine different feature encoding schemes. For each sample, the corresponding amino acid sequence is represented as:

$P=A_{1}A_{2}A_{3}A_{4}A_{5}A_{6}A_{7}A_{8}\ldots A_{L-2}A_{L-1}A_{L}$,

where $A_{i}\left( i=1,2,3,\ldots,L-1,L \right)$ represents the amino acid residue at position *i*.

***K-mer encoding***: This encoding scheme calculates the frequency of all possible sub-sequences of length *k* for each sample. In order to avoid the curse of dimensionality, we set *k* = 1 and 2 to generate 420 features for calculating the frequency of 20 amino acids and 400 amino acid pairs in a protein sequence, respectively.

***DR (Distance-based residues) encoding:*** In this encoding scheme, each protein sequence can be represented as a (20+20*20**d_max_*)-dimensional numerical feature vector based on the distance between amino acid residue pairs, where *d_max_* is the distance threshold representing the maximum distance between residue pairs [1]. For example, if we set *d_max_* = 2, each protein sequence can be represented with an 820-dimensional feature vector (**Figure S1**), consisting of the occurrences of 20 amino acids (*d* = 0), the occurrences of 400 amino acid pairs for two adjacent residues (*d* = 1) and the occurrences of 400 amino acid pairs for residues with distance *d* (*d* = 2).


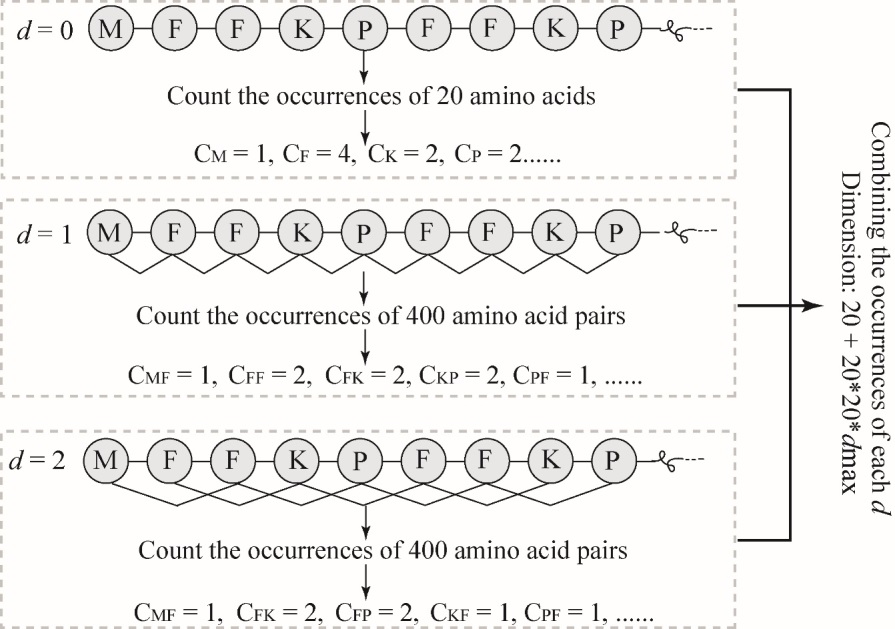


**Figure S1.** The schematic example of ***DR encoding*** scheme

***AC (Auto-covariance) encoding:*** The encoding scheme utilizes various physicochemical properties (PCP) [2] to measure the interactions between *i*-th and (*i+d*)-th amino acid, and has been successfully applied in the prediction of protein interactions [3]. The ***AC encoding*** features are defined as below:

$AC=\frac{\sum_{i=1}^{L-d} \left( P_{u}\left( A_{i} \right)-\bar{P}_{u} \right)\left( P_{u}\left( A_{i+d} \right)-\bar{P}_{u} \right)}{L-d}$,

where $P_{u}\left( A_{i} \right)$ represents the numerical value of the physicochemical index $\mu$ for the amino acid $A_{i}$ and can be accessed from AAindex [2], $\bar{P}_{u}$ is the average value for physicochemical index $\mu$ along the whole sequence and can be calculated as:

$\bar{P}_{u}=\frac{\sum_{i=1}^{L} P_{u}\left( A_{i} \right)}{L}$,

As a result, each protein sequence can be represented as a *N***d*-dimensional numerical feature vector, where *N* is the number of amino acid indexes.

***CC (Cross-covariance) encoding:*** *CC encoding* is another PCP-based feature representation [4, 5], which measures the correlation of two different physicochemical properties between *i*-th and (*i+d*)-th amino acid. Similar to ***AC***, the ***CC***-based feature vector can be calculated as:

$CC=\frac{\sum_{i=1}^{L-d} \left( P_{u1}\left( A_{i} \right)-\bar{P}_{u1} \right)\left( P_{u2}\left( A_{i+d} \right)-\bar{P}_{u2} \right)}{L-d}$ ,

where $P_{u1}\left( A_{i} \right)$ means the numerical value of the physicochemical index $u1$ for the amino acid $A_{i}$ while $P_{u2}\left( A_{i+d} \right)$ means the numerical value of the physicochemical index $u2$ for the amino acid $A_{i+d}$. $\bar{P}_{u1}$ and $\bar{P}_{u2}$ represent the average values for physicochemical index $\mu1$ and $\mu2$, respectively.

***ACC (Auto-cross-covariance) encoding:* *ACC*** is a combination of ***AC*** and ***CC***, which measures both the correlation of two same and different physicochemical properties between *i*-th and (*i+d*)-th amino acid. The length of the ***ACC*** feature vector is *N***N***d*, where *N* is the number of physicochemical indices and *d* is the distance between two residues*.*

***PDT (Physicochemical distance transformation) encoding:*** *PDT* transforms each protein sequence into a fixed-length numeric vector by integrating the sequence-order information with *PCP* [6]. The sequence-order information with physicochemical properties can be represented as:

$$\partial_{d}^{u}=\frac{\sum_{i=1}^{L-d} D_{u}\left( A_{i},A_{i+d} \right)}{L-d}$$

where $d$ is the distance between two amino acids along the protein sequence, and $D_{u}\left( A_{i},A_{i+d} \right)$ can be calculated as:

$D_{u}\left( A_{i},A_{i+d} \right)={(P_{u}\left( A_{i} \right)-P_{u}\left( A_{i+d} \right))}^{2}$,

where $P_{u}\left( A_{i} \right)$ and $P_{u}\left( A_{i+d} \right))$ represent the normalized physicochemical property values (index *u*) of amino acid $A_{i}$ and $A_{i+d}$, respectively. The normalization can be calculated as:

$P_{u}\left( A_{i} \right)=\frac{\hat{P}_{u}\left( A_{i} \right)-\sum_{m=1}^{20} \frac{\hat{P}_{u}\left( A_{m} \right)}{20}}{\sqrt{\frac{\sum_{k=1}^{20} \left( \hat{P}_{u}\left( A_{k} \right)-\sum_{m=1}^{20} \frac{\hat{P}_{u}\left( A_{m} \right)}{20} \right)^{2}}{20}}}$,

where $\hat{P}_{u}\left( A_{i} \right)$ represents the raw physicochemical property value of amino acid $A_{i}$ in index *u*, and $A_{m}(m=1,2,3,4,\ldots,20)$ represents the 20 standard amino acids.

***PC-PseAAC (Series correlation pseudo acid composition) encoding:*** *PC-PseAAC* is another method which incorporates sequence-order information to represent each protein sequence, and is originally introduced for the prediction of protein subcellular localization and membrane proteins [7]. For a given protein with sequence length ***L***, PC-PseAAC can be depicted as:

$\boldsymbol{P}= \left[ p_{1}, p_{2},\ldots, p_{20},\ldots, p_{20+1}, p_{20+2},\ldots, p_{20+\lambda} \right] (\lambda<L)$.

The component in ***P*** is calculated by:

$$P_{u}=\left\{ \begin{aligned} \frac{f_{u}}{\sum_{i=1}^{20} f_{i}+\omega\sum_{k=1}^{\lambda} \tau_{k}}, & 1 \leq u \leq20 \\ \frac{\omega\tau_{u}-20}{\sum_{i=1}^{20} f_{i}+\omega\sum_{k=1}^{\lambda} \tau_{k}}, & 20+1 \leq u \leq20+\lambda\end{aligned} \right.,$$

Where $f_{u}(u=1,2,\ldots,20)$ is the normalized occurrence frequency of 20 amino acids in a protein sequence, $\omega$ is the weight factor and $\tau_{k}$ is the correlation factor that reflects the sequence order correlation between all the *k*-th most contiguous residues as formulated by:

$\tau_{k}= \frac{1}{3(L-k)}\sum_{i=1}^{L-k} \left\{ \left[ H_{1}(A_{i+k})-H_{1}(A_{i}) \right]^{2}+ \left[ H_{2}(A_{i+k})-H_{2}(A_{i}) \right]^{2}+\left[ M(A_{i+k})-M(A_{i}) \right]^{2} \right\}$,

Here $H_{1}(A_{i})$, $H_{2}(A_{i})$ and $M(A_{i})$ represent the value of normalized hydrophobicity, the value of hydrophilicity and the side chain mass for amino acid residue $A_{i}$, respectively, while $H_{1}\left( A_{i+k} \right), H_{2}\left( A_{i+k} \right) andM_{1}(A_{i+k})$ are those for amino acid residue $A_{i+k}$. **Figure S2** shows the details about how to calculate the parameter $\tau_{k}$.


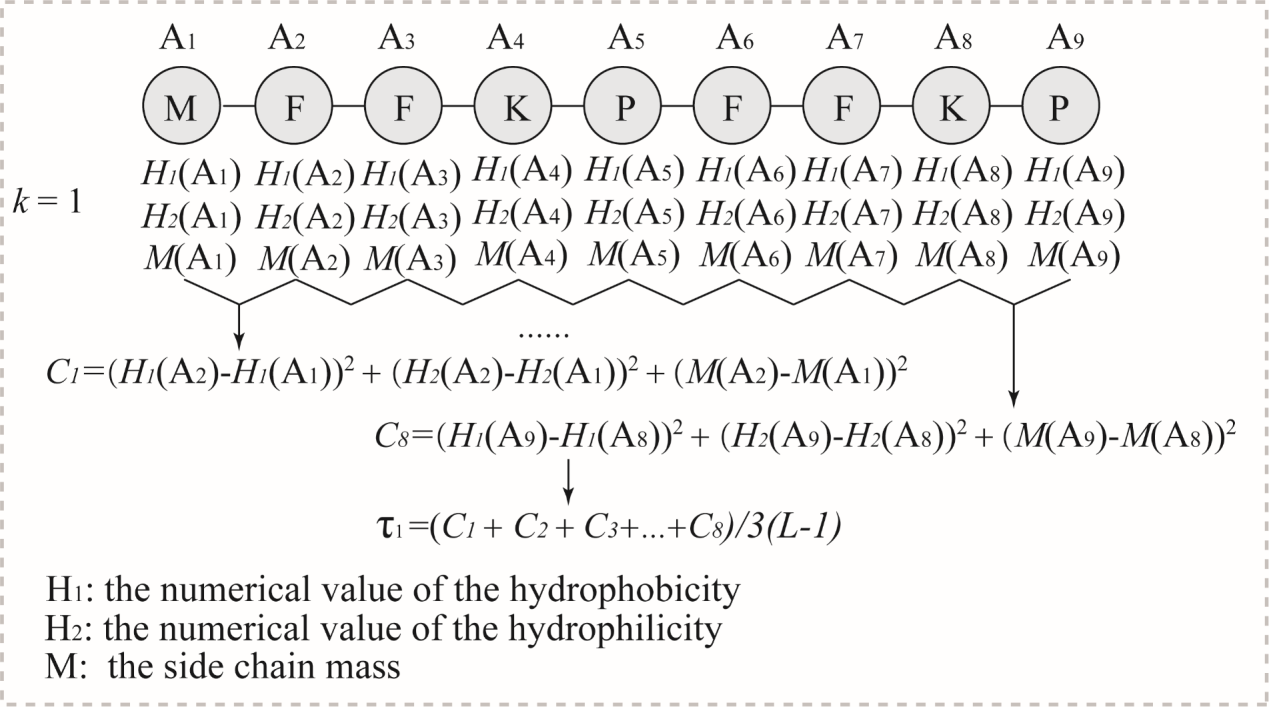
**Figure S2.** The flowchart of calculating the parameter $\tau$.

***SC-PseAAC (General series correlation pseudo amino acid composition) encoding***: *SC-PseAAC*, also named amphiphilic pseudo amino acid composition, is firstly used for predicting enzyme subfamily classes [8]. In this encoding scheme, a given protein ***P*** can be represented as:

$P= \left[ p_{1}, p_{2},\ldots, p_{20},\ldots, p_{20+1}, p_{20+2},\ldots, p_{20+2\lambda} \right](\lambda<L)$,

where the $20+2\lambda$ components are calculated by:

$P_{v}=\left\{ \begin{aligned} \frac{f_{v}}{\sum_{i=1}^{20} f_{i}+\omega\sum_{j=1}^{2\lambda} \tau_{j}}, & 1 \leq v \leq20 \\ \frac{\omega\tau_{v}-20}{\sum_{i=1}^{20} f_{i}+\omega\sum_{j=1}^{2\lambda} \tau_{j}}, & 20+1 \leq v \leq20+2\lambda\end{aligned} \right.$.

The parameter $\tau_{j}$ can be represented as follows:

$\tau_{m}= \frac{1}{L-l}\sum_{i=1}^{L-l} h^{1}\left( A_{i} \right)h^{1}(A_{i+1})$,

$\tau_{n}= \frac{1}{L-l}\sum_{i=1}^{L-l} h^{2}\left( A_{i} \right)h^{2}(A_{i+1})$,

where $m=1, 3, 5, \ldots, (2\lambda-1)$; n$=2, 4, 6, \ldots, 2\lambda$; *l*$=1, 2, 3, \ldots, \lambda$($\lambda<L$); $h^{1}\left( A_{i} \right)$ and $h^{2}\left( A_{i} \right)$ represent the normalized hydrophobicity and hydrophilicity properties of amino acid residue $A_{i}$, respectively; $h^{1}(A_{i+1})$ and $h^{2}(A_{i+1})$ represent the corresponding values of amino acid residue $R_{i+1}$.

***CUB (Codon Usage Bias) encoding***: In this encoding strategy, a log-likelihood ratio score (Hexamer score) in two species was calculated. For the coding sequence of an mRNA with a length of 3**L*, we firstly divided the sequence into hexamers (3**L*-5) by sliding 1bp. Then the hexamer score under a specified species can be calculated as:

$Hexamer score= \frac{1}{3*(L-5)}\sum_{i=1}^{3*(L-5)} log\left( \frac{F\left( H_{i} \right)}{F^{'}\left( H_{i} \right)} \right)$,

where $F\left( H_{i} \right)\left( i=1,2,\ldots,3*L-5 \right)$ and $F^{'}\left( H_{i} \right)\left( i=1,2,\ldots,3*L-5 \right)$ represent the frequencies of *i*-th hexamer in coding sequences and background sequences (genomic DNA sequences), respectively.

- 1. **Predictor construction**

To construct the SAT predictor, each sample (coding sequence) was converted into a 2,257-dimensional numeric vector, and then the generated feature matrix with *M* rows (the number of samples) and *N* (2,257) columns were subsequently fed into the deep forest algorithm to build a predictor to classify transcripts. Deep forest (RF) is a decision tree-based ensemble learning method which harbors the complexity of deep neural network but without hyper-parameter tuning [9].

- 1. **Five-fold cross validation on training samples**

Cross-validation has been widely used to evaluate the performance of ML-based predictors [10-12]. In this study, we used a five-fold cross-validation experiment to evaluate the performance of SAT. In a five-fold cross-validation experiment, positive and negative samples were randomly partitioned into five parts; each part had a roughly equal number and subsequently was used for evaluating the performance of SAT trained with the remaining four parts of samples. In each fold of cross-validation experiment, the receiver operating characteristic (ROC) curve was used to assess the prediction accuracy through plotting the curve of TPR (*y* axis: true positive rate) versus FPR (*x* axis: false positive rate) at all possible thresholds. Then the area under the ROC curve (AUC value) was used to quantitatively score the prediction accuracy of a SAT predictor. An AUC value can range from 0 to 1, and a higher AUC value indicates better prediction accuracy. After assessing AUC with each of the four parts, the mean of the five AUC values was used to represent the overall performance of a SAT predictor.

- 1. **Performance evaluation on the hold-out testing samples**

In order to further assess the performance of SAT, six commonly used measures including Sensitivity (Sn), Specificity (Sp), Precision (Pr), Accuracy (Acc), Matthews correlation coefficient (MCC) and F_1_-score (F_1_) were used to evaluate the performance of SAT predictor on the hold-out testing samples. These measures are defined as follows:

$Sn= \frac{\mathrm{TP}}{TP+FN}$,

$Sp= \frac{\mathrm{TN}}{TN+FP}$,

$Pr= \frac{\mathrm{TP}}{TP+FP}$,

$Acc= \frac{TP+TN}{TP+TN+FP+FN}$,

$F_{1}=\frac{2*Pr\times Sn}{Pr+Sn}=\frac{2*TP}{2*TP+FP+FN}$,

$MCC= \frac{TP*TN-FP*FN}{\sqrt{\left( TP+FP \right)*\left( TP+FN \right)*\left( TN+FP \right)*(TN+FN)}}$,

where TP, TN, FP, and FN represent the number of true positives, true negatives, false positives and false negatives, respectively. F_1_ is the harmonic mean of Pr and Sn. Acc and MCC combine all of the predictions (TP, TN, FP, and FN) into a single score. Acc, which ranges from 0 to 1, measures the proportion of correct predictions. MCC, also known as the phi coefficient, measures the correlation between the observations and predictions. It is generally regarded as a balanced measure, which can be used even if the two classes are of very different sizes. The value of MCC ranges from -1 to 1, where 1 represents a perfect prediction, 0 indicates no better than random prediction and -1 means total disagreement between observations and predictions.

- 1. **Species prediction of newly assembled transcripts**

For each transcript assembled from unmapped reads, SAT assigns a probabilistic score indicating the likelihood that the transcript belongs to a pathogen or a host species.

**2 Experimental validation of newly assembled transcripts**

**2.1 Plant materials and stress treatment**

Maize (*Zea mays* cv. B73) seeds were soaked in deionized water for 12 h and then placed on a sheet of moist filter paper in a Petri dish to allow germination at 28℃ for 3 days. Germinated seeds were cultivated and dehydration stress treatment of 12-day-old maize seedlings was carried out as the method described by Tan *et al* [13]. After the treatment, the shoots and roots of the control and stressed seedlings were harvested in three biological replicates, respectively, immediately frozen in liquid nitrogen, and stored at -80℃ for RNA extraction.

Wheat (Wheat cv. XY 6) seeds were soaked in deionized water for 12 h and then placed on a sheet of moist filter paper in a Petri dish to allow germination at 20℃ for 7 days. Wheat seedling were vernalized at two-leaf stage, and then moved to pots. Leaves at four-leaf stage, roots at three-leaf stage, flag leaves at booting stage, 4cm and 8cm young ears at booting stage, and seeds at 5/10/15/20/25 days of anthesis were harvested in three biological replicates, respectively, immediately frozen in liquid nitrogen, and stored at -80℃ for RNA extraction.

Chinese yellow rust race 31, which have a similar genetic background with CYR32 [14], was used to inoculate the second leaf of wheat seedlings (wheat cv. Shuiyuan 11) at three-leaf stage. The wheat seedlings were then cultivated in soil in a growth chamber at 16℃ under a 16h light/8h dark photoperiod using a paint brush. The wheat seedlings inoculated were then kept wet 24 h, and then harvested in three biological replicates, immediately frozen in liquid nitrogen, and stored at -80℃ for RNA extraction.

**2.2 RNA isolation and cDNA synthesis**

Total RNA was isolated from above plants using TRIZOL reagent (Invitrogen, California, USA). For each sample, 20 μg of total RNA was digested in a volume of 20 μL with RNase-free DNase I (TaKaRa, Dalian, China) according to the manufacturer’s instructions for treatment to remove genomic DNA contamination. The first-strand cDNA synthesis was performed using 5 μg of DNase-treated total RNA with the PrimeScript™ II 1st Strand cDNA Synthesis Kit (TaKaRa, Dalian, China) in a reaction volume of 20 μL following the manufacturer’s instructions.

**2.3 PCR amplification of novel assembled transcripts and sequencing of PCR products**

The specific primers of transcripts selected for experimentally validation were designed using the Primer Premier 5 software [15]. The first-strand cDNA of seedlings of each plant material was used as the template of PCR amplification. PCR amplification of these transcripts was conducted in 20 μL reaction volume by using 250 ng cDNA, 0.2 mM dNTPs, 0.5 lM each primer, 1.5 mM MgCl_2_, PCR buffer and 1.5 U Taq DNA polymerase (a proofreading polymerase; TaKaRa, Dalian, China). PCR cycling was 94℃ for 5 min, followed by 35 cycles of 94℃ for 40 s, 56℃ for 40 s, 72℃ for 1 min, and a final extension at 72℃ for 5 min.

PCR products were separated on a 1.5% agarose gel. The desired sequence fragments were extracted and purified using a gel extraction kit (Omega Bio-Tek, Inc., Doraville, USA), and sequenced by AuGCT Biotechnology Company (Beijing, China).

**References**

1. Liu B, Xu J, Zou Q et al. Using distances between Top-n-gram and residue pairs for protein remote homology detection, BMC Bioinformatics 2014;15 Suppl 2:S3.

2. Kawashima S, Pokarowski P, Pokarowska M et al. AAindex: amino acid index database, progress report 2008, Nucleic Acids Res 2008;36:D202-D205.

3. Zhou C, Yu H, Ding Y et al. Multi-scale encoding of amino acid sequences for predicting protein interactions using gradient boosting decision tree, PLoS One 2017;12:e0181426.

4. Guo Y, Yu L, Wen Z et al. Using support vector machine combined with auto covariance to predict protein-protein interactions from protein sequences, Nucleic Acids Res 2008;36:3025-3030.

5. Dong Q, Zhou S, Guan J. A new taxonomy-based protein fold recognition approach based on autocross-covariance transformation, Bioinformatics 2009;25:2655-2662.

6. Liu B, Wang X, Chen Q et al. Using amino acid physicochemical distance transformation for fast protein remote homology detection, PLoS One 2012;7:e46633.

7. Chou KC. Prediction of protein cellular attributes using pseudo-amino acid composition, Proteins 2001;43:246-255.

8. Chou KC. Using amphiphilic pseudo amino acid composition to predict enzyme subfamily classes, Bioinformatics 2005;21:10-19.

9. Zhou Z-H, Feng J. Deep forest: Towards an alternative to deep neural networks, arXiv preprint arXiv:1702.08835 2017.

10. Cui H, Zhai J, Ma C. miRLocator: Machine learning-based Prediction of mature microRNAs within plant pre-miRNA dequences, PLoS One 2015;10:e0142753.

11. Ma C, Xin M, Feldmann KA et al. Machine learning-based differential network analysis: a study of stress-responsive transcriptomes in *Arabidopsis*, Plant Cell 2014;26:520-537.

12. Ma C, Zhang HH, Wang X. Machine learning for Big Data analytics in plants, Trends Plant Sci 2014;19:798-808.

13. Tan J, Miao Z, Ren C et al. Evolution of intron-poor clades and expression patterns of the glycosyltransferase family 47, Planta 2018; 247:745-760.

14. Chen W, Wu L, Liu T et al. Race dynamics, diversity, and virulence evolution in Puccinia striiformis f. sp. tritici, the causal agent of wheat stripe rust in China from 2003 to 2007, Plant disease 2009;93:1093-1101.

15. Lalitha S. Primer premier 5, Biotech Software & Internet Report: The Computer Software Journal for Scient 2000;1:270-272.
